# Supplementary material for: L-Serine-Modified Poly-L-Lysine as a Biodegradable Kidney-Targeted Drug Carrier for the Efficient Radionuclide Therapy of Renal Cell Carcinoma
Source: Pharmaceutics. 2022 Sep 14;14(9):1946. doi: 10.3390/pharmaceutics14091946 (PMC9503061; doi:10.3390/pharmaceutics14091946)
Supplement: Supplementary file 1 [file pharmaceutics-14-01946-s001.zip › pharmaceutics-1865689-supplementary.pdf]

pharmaceutics

MDPI

Supplementary Materials:

L-Serine-Modified Poly-L-Lysine as a Biodegradable Kidney-Targeted Drug Carrier for the Efficient Radionuclide Therapy of Renal Cell Carcinoma

Hidemasa Katsumi, Sho Kitada, Shintaro Yasuoka, Rie Takashima, Tomoki Imanishi, Rina Tanaka, Satoru Matsuura, Hiroyuki Kimura, Hidekazu Kawashima, Masaki Morishita and Akira Yamamoto

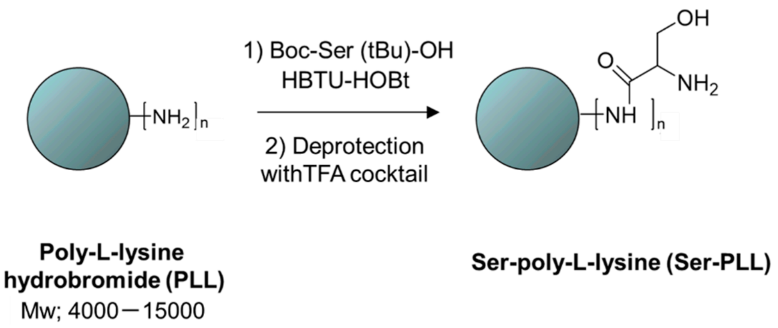

Scheme S1. Ser-poly-L-lysine (Ser-PLL) synthesis.

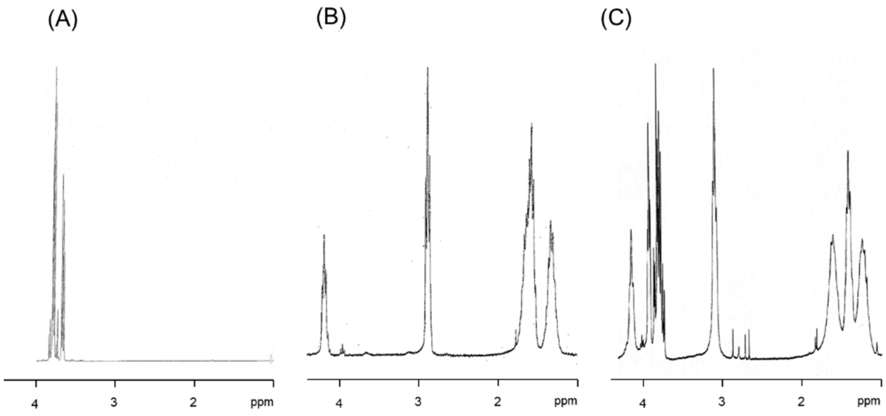

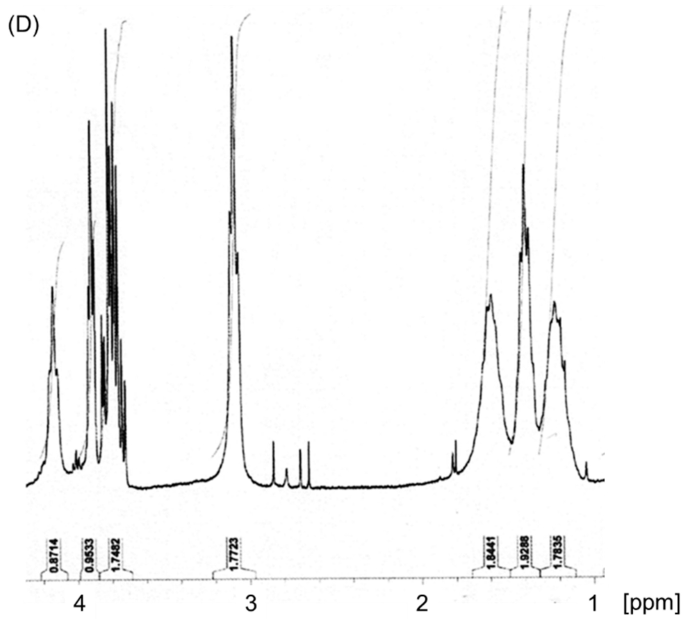

Figure S1. ^{1}H-NMR spectra for Ser (**A**), poly-L-lysine (PLL) (**B**), and Ser-poly-L-lysine (Ser-PLL) (**C**,**D**) in deuterated water.

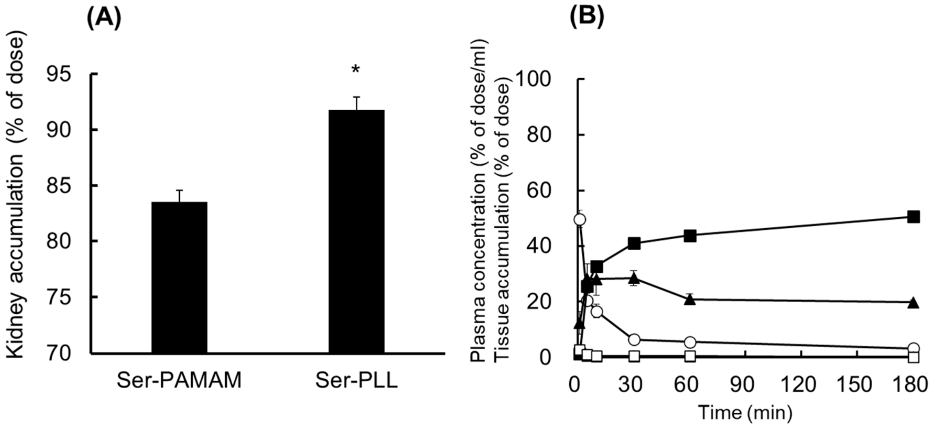

Figure S2. (**A**) Kidney accumulation of ^{111}In-labeled Ser-polyamidoamine (PAMAM) dendrimer (G3) and ^{111}In-labeled Ser-poly-L-lysine (Ser-PLL) after intravenous injection in mice. (**B**) Plasma concentration and tissue accumulation of ^{111}In-labeled high-MW Ser-poly-L-lysine (Ser-PLL(H)) wherein poly-L-lysine (MW range = 15,000–30,000) was conjugated with Ser after intravenous injection into mice. Data are means ± SE for three mice. ◯, plasma; ▲, liver; ■, kidney; ◇, spleen; △, heart; □, lung. * statistically significant difference compared with Ser-PAMAM group ($p < 0.05$).
